# Herbal Medicine and Lifestyle Modifications for People with Obesity: A Single-Center, Retrospective, Observational Study

**DOI:** 10.3390/ph18091396

**Published:** 2025-09-17

**Authors:** Minwoo Bang, Suyong Shin, Jungsang Kim, Minwhee Kang, Donghun Lee, Junho Kim, Chunghee Kim, Jiyoung Son, Seungyeon Choi, Seonghyeon Jeon, Dasol Park, Byungsoo Kang, Jungtae Leem

**Affiliations:** 1 Daeat Korean Medicine Clinic Seoul, 121, Dosan-daero, Gangnam-gu, Seoul 06035, Republic of Korea; bmwdoctor@da-eat.co.kr (M.B.); seungy9497@da-eat.co.kr (S.C.); dm325001@da-eat.co.kr (S.J.); 2 Daeat Research Institute, 121, Dosan-daero, Gangnam-gu, Seoul 06035, Republic of Korea; sjy0425@daeat-ca.co.kr; 3 Daeat Korean Medicine Clinic Incheonbupyeng, 17, Jubuto-ro, Bupyeong-gu, Incheon 21390, Republic of Korea; sypride081@daeat-bp.com; 4 Department of Preventive Medicine, College of Korean Medicine, Kyung Hee University, 26, Kyungheedae-ro, Dongdaemun-gu, Seoul 02447, Republic of Korea; sportplayer1@daeat-sw.com; 5 Daeat Korean Medicine Clinic Gyeonggisuwon, 141, Jungbu-daero, Paldal-gu, Suwon-si 16240, Gyeonggi-do, Republic of Korea; 6 Daeat Korean Medicine Clinic Gyeonggiilsan, 33, Jeongbalsan-ro, Ilsandong-gu, Goyang-si, Gyeonggi-do 10402, Republic of Korea; kmh@daeat-is.co.kr; 7 Daeat Korean Medicine Clinic Busan, 177, Beomil-ro, Busanjin-gu, Busan 47358, Republic of Korea; dh.lee@daeat-bs.co.kr; 8 Daeat Korean Medicine Clinic Daegu, 341, Dongdaegu-ro, Suseong-gu, Daegu 42019, Republic of Korea; dgdaeat@daeat-dg.co.kr; 9 Daeat Korean Medicine Clinic Changwon, 103, Jungdongjungang-ro, Uichang-gu, Changwon-si 51380, Gyeongsangnam-do, Republic of Korea; megachung1@daeat-cw.co.kr; 10 Daeat Korean Medicine Clinic Cheonan, 73-8, Buldang 23-ro, Seobuk-gu, Cheonan-si 31156, Chungcheongnam-do, Republic of Korea; 11 Department of Diagnostics, School of Korean Medicine, Wonkwang University, 460, Iksan-daero, Iksan-si 54538, Jeollabuk-do, Republic of Korea; mare927@naver.com; 12 Department of Ophthalmology, Otolaryngology & Dermatology, College of Korean Medicine, Gachon University, 1342, Seongnam-daero, Sujeong-gu, Seongnam-si 13120, Gyeonggi-do, Republic of Korea; 13 Research Center of Traditional Korean Medicine, College of Korean Medicine, Wonkwang University, 460, Iksan-daero, Iksan-si 54538, Jeollabuk-do, Republic of Korea; 14 Department of Il-won Integrated Medicine, Wonkwang University Korean Medicine Hospital, 895, Muwang-ro, Iksan-si 54538, Jeollabuk-do, Republic of Korea

**Keywords:** weight loss, obesity, integrative Korean medicine treatment, herbal medicine, lifestyle modification

## Abstract

**Objectives**: Conventional Western treatments for obesity are associated with various adverse events (AEs). This study aimed to determine the treatment response and safety assessment of an integrative Korean medicine treatment (IKMT), consisting of herbal medicine (HM) and lifestyle modification (LM), for weight loss (WL) in people with obesity. **Methods**: The electronic medical records of outpatients from July 2021 to May 2023 at a Daeat Korean medicine clinic in Seoul were retrospectively reviewed. A total of 3161 patients were evaluated using bioelectrical impedance analysis (BIA) and blood pressure (BP) index. Moreover, the treatment response to IKMT in the 24 best cases (WL within BMI < 23 kg/m^2^) was evaluated using BIA and BP index, and the safety profile was determined by analyzing AEs. **Results**: The mean age was 38.2 ± 11.39 years, and the mean duration of treatment was 142.62 ± 104.92 days (approximately 20 weeks). The mean WL was 8.02 ± 6.67 kg (change from the baseline, 8.71%). Of the 3161 participants, 2146 had a WL of ≥5%. The best-case subgroup (*n* = 24; age 36.54 ± 11.64 years) achieved 23.02 ± 4.07 kg WL and reached BMIs < 23 kg/m^2^ in 7.83 ± 2.54 months; among those with BP indices available (*n* = 21), reductions were statistically significant. In this subgroup, the mean treatment duration was 8.71 ± 2.46 months (range, 5–15), exceeding the 6-month safety guideline for Ephedrae Herba-containing HM, and no serious AEs were observed. At the 7-month follow-up, 11 patients maintained a statistically significant WL. **Conclusions**: This is the first Korean study to apply the professional collaboration of IKMT and dietician-led LM to people with obesity. IKMT combined with LM appears to be a safe and effective approach for obesity management. Prospective studies are needed to confirm these findings and establish standardized treatment protocols.

## 1. Introduction

The World Health Organization (WHO) defines overweight and obesity as a body mass index (BMI) of ≥25 kg/m^2^ and ≥30 kg/m^2^, respectively, for adults [1,2]. Obesity has become significantly prevalent in the global population, and has begun to replace undernutrition and infectious diseases as the leading cause of disease [1]. Complications of obesity include diabetes mellitus, hypertension, heart disease, obstructive sleep apnea, asthma, nonalcoholic fatty liver disease, osteoarthritis, and polycystic ovary syndrome [3], all of which reduce life expectancy [2] and quality of life [4]. People with obesity (PwO) have higher societal costs than those with a normal body weight (BW) [2], with the global healthcare costs of obesity estimated at USD 2 trillion [5] and projected to cause a 3.3% reduction in the gross domestic product [2].

Phentermine, an anti-obesity medication (AOM), is widely used; however, only short-term use of phentermine, amfepramone, and cathine hydrochloride is recommended owing to their potential for addiction and anemia development [6]. Since 2019, bariatric surgery has been covered by the Korean National Health Insurance Service for PwO having a BMI ≥ 30 kg/m^2^ with certain complications [7]. However, adverse events (AEs) associated with bariatric surgery can be extensive and serious, including fractures, adverse maternal and fetal outcomes (postnatal mortality, genital abnormalities, preterm birth, and intrauterine growth restriction), and mental health outcomes (including suicide, self-harm, and alcohol use disorders) [8]. Glucagon-like peptide-1 receptor agonists (GLP-1 RAs) are also popular [9,10,11], but they are also associated with serious AEs like gastrointestinal disorders, hypotension, syncope, arthritic disorders, nephrolithiasis, interstitial nephritis, and drug-induced pancreatitis [12].

Integrative Korean medicine treatment (IKMT) for obesity is widely used in East Asia [13,14]. IKMT generally focuses on clinical symptoms and signs, such as general physical condition, digestive symptoms, pain, and sleep habits, which can minimize the side effects of pharmacological treatment and bariatric surgery [15]. Moreover, IKMT has minimal side effects, and weight loss (WL) can be safely achieved [16]. Randomized controlled trials (RCTs) of herbal medicine (HM), including Ephedrae Herba (EH) at an ephedrine dose of 20–90 mg/day, have been conducted and have shown WL treatment response and safety [17]. However, these studies were conducted in an RCT setting and may not be applicable to the population. The number of real-world data (RWD) studies in clinical practice is small, and even when available, they are only case series [18,19]. Thus, there is a lack of information on the success rate of treatment in clinical practice. However, Daeat Korean medicine clinics (KMCs) provide HM, lifestyle modification (LM), pharmacopuncture (PA), thread embedding acupuncture (TEA), detoxification therapy (detox), and therapeutic devices for PwO through IKMT, often achieving a WL of >5%, which is the goal of obesity treatment.

In this study, we aimed to perform a retrospective chart review analysis of PwO with BMI ≥ 30 kg/m^2^ in a single-center KMC using IKMT to investigate the treatment success rate in PwO in a real clinical setting, analyze the best patients (WL with BMI < 23 kg/m^2^) who were successfully treated, assess whether they regained BW after a certain period after the end of treatment, and investigate the treatment response and safety of the successful group. This study provides evidence for developing new treatments and improving outcomes in obesity, a disease with limited clinical effectiveness of existing treatments.

## 2. Results

### 2.1. Characteristics of Patients

**Figure 1:**
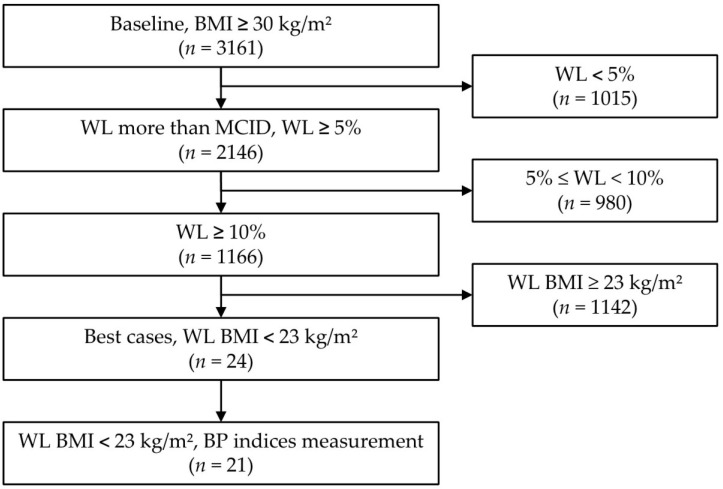
Flowchart of participants according to degree of weight loss. Abbreviations: BMI, body mass index; WL, weight loss; MCID, minimal clinically important difference; BP, blood pressure.

The mean age of the 3161 participants was 38.2 ± 11.39 (range 9–90) years, the mean height was 164.71 ± 8.82 (range 121.70–197.30) cm, and the mean time between the baseline and last bioelectrical impedance analysis (BIA) measurement was 142.62 ± 104.92 (range 7–600) days (approximately 20 weeks). Among the participants, 2238 (70.8%) were female (Figure 1 and Table 1).

### 2.2. Characteristics of the Best-Case Participants

The mean age of the 24 best-case participants was 36.54 ± 11.64 (range, 20–60) years, the mean height was 161.15 ± 7.08 (range, 147.2–179.3) cm, and the mean time between the first and last BIA measurement was 261.75 ± 75.31 (range, 150–450) days. Among the participants, 23 (95.8%) were female (Table 2). Regarding previous treatment for obesity, six (25%) participants had been treated with HM, six (25%) with conventional Western medicine, and two (8.33%) with a combination of both (Table 2). Additional characteristics are listed in Table 2.

The mean time required for BMI reduction to reach a normal range for the 24 most successful cases was 7.83 ± 2.54 (range 5–15) months, with a total treatment duration of 8.71 ± 2.46 (range 5–15) months. The number of face-to-face treatment (FTF) sessions was 7.33 ± 2.37 (3–11), whereas the number of non-FTF (NFTF) sessions was 2.38 ± 2.61 (0–9). Overall, 9 (37.5%) received more FTF sessions than NFTF sessions, whereas 15 (62.5%) received more NFTF sessions than FTF sessions. Of the 15 NFTF-dominant patients, 7 (29.17% of the total) had predominantly NFTF sessions. The Pearson correlation coefficient between age and the percentage of NFTFs was low and not statistically significant (*r* = 0.016, *p* = 0.94).

### 2.3. Treatment Analysis

#### 2.3.1. Herbal Medicine

The HM decoction is a personalized medication prescribed on an individual basis after diagnosis and was administered to 20 (83.33%) of the best cases. Four patients received only the solid dosage form. Of the 20 participants who received HM decoctions, 3 (12.5%) received ≥3 different prescriptions over time, 10 (41.67%) received 2 prescriptions, and 7 (29.17%) received 1 prescription (Table 3).

The basic decoction of Daeat KMC, known as Daeat-tang, consists of the following ingredients: EH, Cinnamomi Ramulus, Zizyphi Fructus, Rhei Radix et Rhizoma, Atractylodis Rhizoma Alba, Poria Sclerotium, Gypsum Fibrosum, Bupleuri Radix, Arctii Fructus, Coicis Semen, Paeoniae Radix, Alismatis Rhizoma, Magnoli-ae Cortex, Cyperi Rhizoma, Zingiberis Rhizoma Recens, and other ingredients. Among the various solid dosage forms (including pills, tablets, and capsules), Daeat-dan (pill) was prescribed to all 24 patients, whereas tablets and capsules were prescribed to 16 and 4 patients, respectively.

#### 2.3.2. Other Treatments

PA was used in 11 (45.8%) patients with LIPOSA-S alone and in 2 (8.3%) patients with LIPOSA-S and Bamboo Salt. The PA treatment sites included the abdomen and thighs. TEA was administered to the abdomen in six (25%) patients. All 24 patients underwent detox before treatment. Eighteen (75%) and 20 (83.33%) participants received therapeutic device treatment and Daeat close coaching, respectively.

### 2.4. Weight Reduction Effect

**Figure 2:**
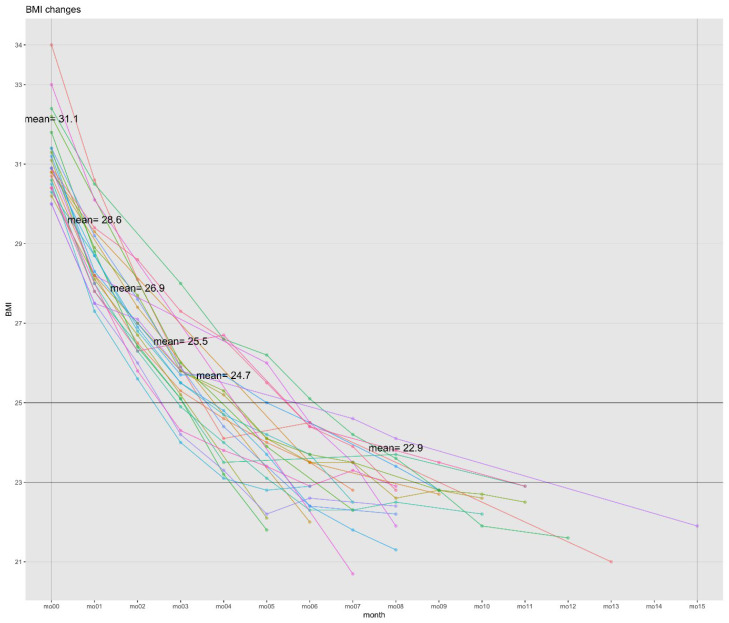
BMI changes in best cases (*n* = 24). Abbreviations: BMI, body mass index. Best cases: weight loss with a BMI < 23 kg/m^2^.

A paired *t*-test was performed to determine the discrepancy between each item in the patients’ pre- and post-treatment BIA results. The baseline and endpoint BIA measurements were compared. The final in-person visit was used as the endpoint, because some patients underwent NFTF. The BIA changes for the 3161 patients and 24 best cases are shown in Table 4 and Table 5, respectively. In the best cases, the WL was 28.42%. In addition, the WL of 3161 individuals exceeded the minimal clinically important difference (MCID) of 2.5 kg for BW (8.02 ± 6.67 kg) and exceeded the MCID of 5% for percent body WL (%WL; 8.71%), both of which were statistically significant. The reductions in BMI were 8.90 ± 1.47 kg/m^2^ in the best cases and 2.96 ± 2.41 kg/m^2^ in 3161 cases, both of which were statistically significant (Figure 2, Table 4 and Table 5). Additionally, statistically significant reductions in total body water (TBW), body fat mass (BFM), body fat percentage (BF%), waist-to-hip ratio (WHR), and Broca’s index (BI) were observed (Table 4 and Table 5). In the best cases, the values for BMI, WHR, and BI fell below the established cutoff values of 23 kg/m^2^, 0.85, and 120, respectively [20,21].

At the 7-month follow-up, 11 of the 24 patients were followed up. A paired *t*-test showed that the change in BW between the end of treatment and follow-up 7 months later was 1.21 ± 5.16 kg, which was not a statistically significant increase (*p* = 0.455), whereas WL between baseline and follow-up 7 months later was 19.76 ± 5.25 kg, which was a statistically significant WL (*p* < 0.001). At follow-up, WL was 24.87 ± 0.06% from baseline, and BMI decreased by a mean of 7.65 ± 1.99 kg/m^2^, which was statistically significant (*p* < 0.001).

### 2.5. Index of Blood Pressure

Obesity is associated with high blood pressure (BP) and pulse pressure (PP) [22]. High BP and PP are significant predictors of myocardial infarction, stroke, and other cardiovascular events [22]. Of the 3161 participants, 1869 were comparable before and after BP index assessment (Table 6). Additionally, of the 24 best cases, 21 were comparable before and after BP index assessment (Table 6). Only comparable participants were subjected to paired *t*-tests. The changes in systolic blood pressure (sBP), diastolic blood pressure (dBP), mean blood pressure (mBP), and PP were statistically significant, whereas the heart rate (HR) showed an increase, but was not statistically significant in the best cases (Table 6).

### 2.6. Safety Assessment

AEs identified in previous studies were categorized into 27 items, including other items (Table 7). A total of 55 AEs were reported in 20 patients, with constipation being the most common in 17 (70.83%) cases, followed by nausea in 7 (29.17%), dry mouth in 6 (25%), dizziness in 6 (25%), and insomnia in 4 (16.67%). All AEs were classified as mild in severity, with 6 (10.9%) classified as possible and 49 (89.1%) as unlikely in terms of causality. All AEs improved with the active HM treatment, including the prescription of additional pills, changes in the composition of the HM decoction, and adjustments in the dose of EH in solid HM. Most AEs resolved within 3–14 days after these interventions, and no patient reported any self-medication during the study period.

## 3. Discussion

### 3.1. Summary of Findings

From July 2021 to May 2023, 3161 PwO with a BMI of ≥30 kg/m^2^ visited the Daeat KMC. The mean duration of treatment was 142.62 ± 104.92 (7–600) days, which corresponds to approximately 20 weeks. Of these, 2146 (67%) patients demonstrated an MCID of ≥5% at the final visit. In addition, statistically significant reductions in BP indices, including sBP, dBP, mBP, and PP, were observed, suggesting improvements in cardiovascular risk indicators. Of the 24 best cases, 11 were contacted by telephone 7 months after the end of treatment. The results of the paired *t*-test showed that the change in BW between the end of treatment and the 7-month follow-up was 1.21 ± 5.16 kg, which was not statistically significant (*p* = 0.455). Conversely, the BW change between the baseline visit and the 7-month follow-up visit was –19.76 ± 5.25 kg, which was a statistically significant WL (*p* < 0.001).

A BMI of <23 kg/m^2^, which is considered normal according to Korean standards, was defined as the best case, at the end of treatment, and 24 patients were selected for follow-up to assess the incidence of BW regain. In addition, 20 (83.33%) patients who were administered a solid dosage form containing EH achieved WL using various medications tailored to their individual constitutions and health status. We identified a successful and safe treatment course for PwO with BMI ≥ 30 kg/m^2^, suggesting that IKMT may be a promising initial treatment option for those who have experienced difficulty achieving WL with nondrug treatments or who do not require bariatric surgery. This was the first retrospective case series in Korea to apply IKMT and professional collaboration for LM by a registered dietitian in PwO.

### 3.2. Comparisons with Previous Studies

At 3–6 months in RWD, 14–58.6% of patients treated with orlistat, phentermine/topiramate, naltrexone/bupropion, phentermine, or liraglutide achieved a WL of ≥5% [23]. However, longer RCTs (56–72 weeks) of GLP-1 RAs reported ≥5% WL rates of 63.2% (liraglutide), 86% (semaglutide), and 91% (tirzepatide) [9,10,11]. In this study, IKMT at Daeat KMC resulted in a WL success rate of 67.9%, with an average treatment duration representing only 36% and 28% of the treatment durations for liraglutide and tirzepatide, respectively.

Importantly, the differences in WL rates and mean percentage WL between studies are largely attributable to variations in treatment duration and study design, specifically RWD versus RCTs. The RWD studies include cases with poor medication adherence (MA), whereas the GLP-1 RA RCTs generally do not, which limits the direct comparability of treatment responses. For example, the mean percent WL from baseline at 142.62 ± 104.92 days (approximately 20 weeks) in RWD was 8.71%, compared with 7% (liraglutide), 9% (semaglutide), and 12% (tirzepatide) in non-RWD settings [9,10,11]. Additionally, in South Korea, WL at 6 months based on RWD was 5.9% (liraglutide) and 7.7% (phentermine/topiramate; Qsymia) [24].

### 3.3. Characteristics, Advantages, and Clinical Perspective of Integrative Korean Medicine Treatment

The advantage of IKMT is that WL is not limited to the effects of a single herb, EH, which stimulates the sympathetic nervous system, increases heat production and metabolism, inhibits cholesterol absorption, and increases adipose tissue energy expenditure [17]. Instead, it uses tailored prescriptions for different types of PwO, including those for primary and secondary obesity.

It can be postulated that most patients were female (23 of 24 in the best cases) because obesity clinics have a higher proportion of female patients than male patients. In contrast, male patients tended to be satisfied with their WL and did not strive to lose more BW. The implementation of the NFTF during the ongoing COVID-19 pandemic presents a challenge in maintaining consistent BW measurements at regular intervals via telemedicine. All BIA items, including BMI, showed statistically significant reductions, with a total WL of 28.4%, which exceeded the WHO-recommended WL range of 5–10%. Because the study participants were enrolled in a WL program with a BMI of ≥30 kg/m^2^ and a BMI of ≤23 kg/m^2^, it was expected that all BIA components would show statistically significant changes. Further studies are required to identify the participant selection criteria based on variables other than BMI loss. The reduction in protein mass relative to that in BFM was 4.65%, indicating that the majority of the WL was due to a reduction in BFM.

Manual acupuncture, auricular acupuncture, electroacupuncture, PA, and TEA are effective in treating overweight or obesity by suppressing appetite and reducing hunger and fatigue during WL; when combined with LM, the WL effect can be maximized [15]. In this study, 13 patients with PA, 6 with TEA, and 20 with LM were treated with HM. However, statistical comparison within this group has limited significance because it was conducted only among the best cases, and treatment outcome data for all 3161 patients were not available. Therefore, a direct comparison of the effects of different treatment methods was not possible. Further studies analyzing weight loss effects according to various treatment combinations are warranted.

### 3.4. Safety

No additional AEs occurred beyond those previously documented, and all AEs were mild, suggesting that IKMT is safe for long-term use. The Society of Korean Medicine for Obesity Research recommends the use of dry EH at a dosage of 4.5–7.5 g/day for up to 6 months [25]. Although the average treatment duration in this study exceeded the guideline with 8.71 ± 2.46 (range 5–15) months, no severe AEs were observed. HR increased significantly by 8.11 ± 14.47 bpm overall, from 91.73 ± 13.75 to 99.84 ± 14.34 bpm, but in the optimal subgroup (*n* = 24), the increase of 3.38 ± 12.95 bpm was not statistically significant. Monitoring HR is thus recommended in patients showing significant increases during WL treatment [26]. Potential AEs were minimized by baseline contraindication screening and regular BP monitoring. As clinical experience with long-term EH use grows beyond recommended durations, safety criteria may be reconsidered. However, this study found no AE of long-term EH use on BP indices.

Compared to GLP-1 RAs, where 7.0–9.9% of patients discontinued due to AEs, and 78.9–89.7% reported at least one AE [9,10,11], the optimal IKMT subgroup showed an AE incidence of 83%, similar in frequency but generally mild in severity. Notably, GLP-1 RAs such as liraglutide and tirzepatide have been associated with serious AEs, including cholelithiasis requiring surgery and 6.3% serious AE rates, respectively. Therefore, IKMT at Daeat KMC may offer a safer profile for WL compared to GLP-1 RAs. Future IKMT studies should include patients with higher MA to further validate safety and effectiveness.

### 3.5. Suggested Mechanisms

There are several potential mechanisms underlying the role of HM in obesity. These include the inhibition of endoplasmic reticulum stress, increased leptin sensitivity, and decreased proinflammatory cytokine expression in the liver and adipose tissue in a dose-dependent manner, involving the regulation of lipid metabolism and anti-inflammatory effects, decreasing serum free fatty acids, and ameliorating glucose intolerance/insulin resistance [27].

The mechanism of WL through LM involves changes in diet composition, timing, food type, and physical activity. These changes increase satiety, energy expenditure, and eating behavior, leading to WL. Special diets can affect appetite-regulating hormones, increase postprandial satisfaction, reduce energy intake, and improve metabolism, thereby promoting WL [28].

We hypothesized that the interventions of interest—namely, IKMT (including HM) and LM (including nutritional counseling by a dietitian)—may have influenced WL through these mechanisms. Research on the mechanisms of herbal medicine for obesity treatment increasingly involves not only traditional experimental studies but also the adoption of Network Pharmacology (NP) methodologies [29]. This approach facilitates easier prediction of mechanisms and validation through experiments. It is anticipated that future research on the mechanisms of IKMT will also benefit from the introduction of NP methodologies, enabling more accurate studies of its mechanisms.

The weight-loss mechanisms of the herbal medicines used at Daeat KMC differ from those of conventional anti-obesity drugs, which primarily act through central appetite suppression or nutrient absorption inhibition [6,27]. The herbal prescriptions in this study act via multi-target pathways, including modulation of lipid metabolism, improvement of insulin resistance, anti-inflammatory effects in adipose tissue, and enhancement of thermogenesis [27,29]. According to network pharmacology analyses, key herbs frequently used in these prescriptions—EH, Gardeniae Fructus, Glycine Semen Preparata, and Phellodendri Cortex—contain bioactive compounds such as evodiamine, berberine, genipin, and quercetin, which target adipocytokine signaling, β-adrenergic receptor-mediated lipolysis, thermogenesis, and insulin signaling [29]. Given these mechanistic differences, future anti-obesity drug research should also examine the unique pathways through which herbal medicines exert their effects. Furthermore, pharmacokinetic (PK) studies are essential to elucidate the absorption, distribution, metabolism, and excretion of active herbal components, which will facilitate optimization of dosing regimens, enhance therapeutic efficacy, and minimize adverse events [30]. Such integrated mechanistic and PK research could provide novel therapeutic targets and support the development of safe, effective, and complementary obesity treatments.

### 3.6. Strengths and Limitations of This Study and Suggestions for Future Studies

This study has several notable strengths, including a long-term follow-up period of 7 months after completion of treatment, the inclusion of RWD for PwO with a BMI ≥ 30 kg/m^2^, and the availability of a substantial amount of patient information (>3000 records). However, this study has some limitations. Unlike previous studies on novel GLP-1 RAs, this investigation represents a retrospective RWD of 3161 patients. The assessment of factors influencing the treatment response and AEs was based on a best-case scenario. In addition, this study lacked a placebo control group, an identification of differences in characteristics between responders and nonresponders, and a comprehensive follow-up period. Furthermore, the effect of topical treatments, such as PA and TEA, on localized fat loss was not evaluated using circumference measurements. Additionally, LM was not evaluated as an additional scale. Moreover, potential confounding factors that might have influenced weight loss outcomes—such as patient compliance with herbal medicine prescriptions, the extent of engagement with the mobile nutrition coaching application, and the presence of underlying medical conditions—were not assessed in this study, which should be considered when interpreting the results.

Nevertheless, even when the WL results included data from patients with low MA, the results were comparable to those of GLP-1 RAs. From a clinical perspective, this represents a viable alternative for patients who are unable to adhere to GLP-1 RAs because of AEs or cost considerations or who decline treatment owing to concerns about weight cycling. To address the limitations of the current study, which had a relatively short treatment duration of 142.62 ± 104.92 days (approximately 20 weeks) compared to the 56–72 weeks observed in GLP-1 RA trials, we plan to conduct a larger study with a longer duration of at least 52 weeks. This will include a study design that exclusively enrolls patients with high MA, a placebo-controlled study, and an additional component that includes AE monitoring and blood testing to determine the safety profile of IKMT. In addition, we plan to incorporate circumference measurements or quantitative scales to improve the accuracy of our assessments.

## 4. Materials and Methods

This was a retrospective, single-center study in which the medical records of PwO were analyzed to observe treatment response and safety through assessments such as BIA before and after IKMT. This study was approved by the Daeat KMC Institute Review Board (IRB) (approval number: DIRB-202406-01) as a retrospective chart review, with exemption from review and the requirement for consent. All data were anonymized.

### 4.1. Participants

The inclusion criteria were as follows: first visit to KMC between July 2021 and May 2023, BMI ≥ 30 kg/m^2^ at baseline, and adherence to the Daeat KMC’s WL program.

The exclusion criteria were as follows: inability to compare BMI before and after, concurrent treatment with other medical institutions (including appetite suppressants, liposuction, bariatric surgery, and injectable lipolysis) during IKMT, and any of the Daeat KMC’s initial prescription contraindications (see Table 8).

WL resulting in BMI < 23 kg/m^2^, which is considered a normal weight according to Korean standards, was defined as the optimal outcome.

### 4.2. Treatments

All patients in this study received a standardized core treatment protocol consisting of HM, LM, and detox. Additional optional therapies—such as PA, TEA, and therapeutic devices—were provided only upon patient request or clinical indication and were not mandatory components of the treatment protocol.

#### 4.2.1. Herbal Medicine

The solid formulation of HM was administered thrice a day, whereas the decoction was administered twice a day (morning and evening). The dose was individually prescribed according to the patient’s constitution and health status. The basic ingredients of the pills (pellets) (trade name: Daeat-dan) are presented in Table 9 [31]. There were five different dosage levels, which were determined based on the EH content. In addition to pills, solid dosage forms, such as tablets and capsules, were prescribed depending on the patient’s condition and preference. For weight loss management, Korean medicine doctors (KMDs) at the Daeat KMCs recommended a minimum intake period of 3 months of HM. They also informed patients that, in the case of HM containing EH, the maximum recommended duration of use is 6 months, in accordance with safety guidelines [25].

#### 4.2.2. Lifestyle Modification (Daeat Close Coaching)

LM is strongly recommended as it facilitates WL and maintenance [32]. LM in combination with pharmacotherapy is more effective than pharmacotherapy alone in facilitating WL and improving treatment responses in patients receiving maintenance therapy [33]. In addition, individualized nutritional counseling provided by a dietitian can improve WL outcomes [34]. In this study, LM counseling was provided by a dedicated team of coaching professionals in collaboration with KMDs. Close coaching is a specialized coaching service tailored to each client’s individual needs and lifestyle. This service is provided by a dedicated team of experts, including dietitians. The program is designed to analyze dietary and lifestyle habits through questionnaires and provide ongoing motivation and emotional support before recommending a low-carbohydrate diet and nutrition coaching. The coaching services are further enhanced by a mobile application, the Daeat mobile application (available on Android and iOS), which provides a food awareness and physical activity logbook and was offered to all patients for 2–8 weeks, depending on patient preference.

#### 4.2.3. Pharmacopuncture

PA for obesity was provided only to patients who requested this treatment and were deemed clinically appropriate by a KMD in the absence of contraindications. LIPOSA-S PA (The Academy of Convergence Korean Medicine, Seoul, Republic of Korea), whose main ingredients are Astragali Radix, Pinelliae Tuber, Taraxaci Herba, and Bamboo Salt PA (3%) (Jayeonsaeng Herbal Dispensary, Yongin, Republic of Korea), whose main ingredient is Bamboo Salt, were used. For large areas, such as the abdomen, LIPOSA-S PA 4–8 mL and Bamboo Salt PA 10–20 mL were used, and for relatively small areas, such as the thighs and arms, LIPOSA-S PA 2–4 mL and Bamboo Salt PA 5–10 mL were used. The treatment was recommended to be applied to the same area once a week. PA was provided in the hospital setting only to patients who selected this program component.

#### 4.2.4. Thread Embedding Acupuncture

The threads used for TEA were MONO 27G 38 mm (PINE BM Co., Ltd., Daejeon, Republic of Korea) and MONO 30G 25 mm (DERMALIN Co., Ltd., Hanam, Republic of Korea). For each TEA treatment, 20 threads of the 38 mm product were used for larger areas, such as the abdomen, whereas 10 threads of the 25 mm product were used for smaller areas, such as the thighs and arms. It was recommended that the same area be treated once a week. TEA was performed in the hospital setting for patients opting for this intervention.

#### 4.2.5. Detoxification Therapy

Detox is an herbal detoxification method that aids in rapid WL and the formation of ketone bodies by removing toxins and wastes accumulated in the body. It was administered for 3 days and recommended before starting HM or when WL was slow.

The detox consists of Daeat Haedok-dan, Daeat Bium-dan, and Daeat Sunsik (Misu). The main ingredients of Daeat Haedok-dan are EH, Rehmanniae Radix Preparata, Coicis Semen, Cannabis Semen, Gypsum Fibrosum, Poria Sclerotium, and Sennae Folium. The main ingredients of Daeat Bium-dan are Rhei Radix et Rhizoma, Natrii Sulfas, Cannabis Semen, Armeniacae Semen, Paeoniae Radix, Ponciri Fructus Immaturus, and Magnoliae Cortex. Daeat Sunsik (trade name: Daeat Balance) includes Isolated Soy Protein, Whey Protein Isolate Powder, Lecithin Powder, Enzymatically Modified Stevia, etc. It replaced the regular meals during the detox. Daeat Haedok-dan was administered thrice a day with water, starting with half a serving as the first dose. It was recommended to take the Daeat Sunsik with water, and the Daeat Haedok-dan and Daeat Sunsik can be taken in any order. Daeat Bium-dan was taken once before going to bed, again starting with half a serving. All patients were prescribed the detox regimen during their clinic visit and self-administered it at home.

#### 4.2.6. Therapeutic Device

Noble Shape (Eunsung Global Co., Ltd., Wonju, Republic of Korea) simultaneously irradiates a 658 nm ± 10% low-level laser, amplitude-modulated medium and low frequency of 1–4000 Hz, and radiofrequency of 1 MHz ± 20% noninvasively for approximately 30 min. The 658 nm low-level laser destroys fat cells by liquefying them while creating temporary holes in the fat cell membrane [35]. Amplitude-modulated medium and low frequencies stably stimulate lipolytic biological mechanisms in the human body through current modulation of 1–4000 Hz [36]. The deep heat generated by radiofrequency energy induces fat cell reduction and body fat degradation [37]. Therapeutic devices were mainly applied to large areas, such as the abdomen and, if desired, to other areas, such as the thighs and arms. Treatment was recommended to be applied to the same area once a week. Therapeutic devices were implemented in the hospital setting for those who chose to participate in this program element.

### 4.3. Outcome Measurements and Data Collection

Treatment response was evaluated by analyzing BIA and variations in BP index. Safety was assessed by evaluating AEs that occurred before and after drug administration. A follow-up telephone survey was conducted on 19 January 2024, and only BMI and BW were evaluated. The best-case participants were evaluated for factors such as alcohol consumption, menopausal status, current and past medical history, family history, previous WL treatment, and occupation.

The primary outcome was the change in BMI and BW from baseline to the last visit, as determined using BIA. However, because this was a retrospective chart review rather than a prospective study, the interval between treatments varied among patients. Accordingly, this study extracted and analyzed the results of baseline and follow-up measurements taken immediately after treatment completion. The secondary outcomes included changes from baseline to the last visit in TBW, BFM, BF%, WHR, BI, sBP, dBP, mBP, HR, and PP. PP is the difference between sBP and dBP.

BIA was assessed using InBody370S (InBody, Inc., Seoul, Republic of Korea), BP index was assessed using BPBIO320 (InBody, Inc.), and the data were automatically stored in the cloud (LookinBody; InBody, Inc.) and downloaded in Excel format. BMI and BW were assessed at baseline and subsequent visits. The MCID for percent weight loss was 5% [38], whereas the MCID for BW was 2.5 kg [39].

A follow-up phone call was made on 19 January 2024 to inquire about post-treatment follow-up and BW for the participants with the best results. The participants were asked by phone about their most recent BW.

### 4.4. Statistical Analysis

The R software version 4.3.2 (R Studio, Boston, MA, USA) was used for statistical analyses, and the level of statistical significance for all statistical analyses was set at *p* < 0.05.

#### 4.4.1. Descriptive Statistics Summary of Participants’ Characteristics

Descriptive analyses were performed on the baseline characteristics of the data collected from the study participants. For continuous variables at baseline among PwO with BMI ≥ 30 kg/m^2^, the mean and standard deviation are presented, along with the maximum and minimum values, to illustrate the distribution. Categorical variables are presented as frequencies and proportions.

#### 4.4.2. Evaluation Criteria and Methodology of Treatment Response

To determine the treatment response, BIA is expressed as a continuous variable and subjected to a paired *t*-test analysis of changes from baseline to follow-up (Wilcoxon signed-rank test if not normally distributed). The determination of response and nonresponse was based on the MCID. The proportion of patients in the response group with values above the MCID was examined for each symptom group.

#### 4.4.3. Evaluation Criteria and Methodology of Safety

A comprehensive safety assessment was conducted for all recorded AEs throughout the treatment period to provide a detailed account of AE incidence. The AEs associated with HMs in obesity are primarily related to the cardiovascular or autonomic nervous system. These include increased BP, arrhythmias, palpitations, insomnia, dizziness, headache, sweating, fatigue, dyspepsia, and anxiety neurosis [40]. AEs were documented by patient self-report of symptoms at each outpatient visit or telephone consultation or by investigator observation and BP indices checks at each visit.

The clinical signs of AEs were based on the system organ classes of the WHO Adverse Reactions Terminology. The causality of AEs was classified as certain, probable/likely, possible, unlikely, conditional/unclassified, or unassessable/unclassifiable according to the WHO–Uppsala Monitoring Centre criteria [41]. The severity of AEs was described as grade 1–5 according to the Common Terminology Criteria for Events version 5.0, with increasing severity as the grade number increases [42]. AEs were descriptively reported, and the number of events was totaled and presented. Three KMDs adjudicated the outcomes, with disagreements discussed, and majority rule was applied when at least two of the three adjudicated outcomes were equal.

## 5. Conclusions

This study of IKMT (a combination of HM, LM including PA, TEA, detox, and therapeutic device) in a Daeat KMC has the advantage of being an RWD, despite the limitations of a retrospective study. It showed a clinically meaningful WL rate, with 67.9% achieving ≥5% at 142.62 ± 104.92 days (approximately 20 weeks) in a large RWD study. However, direct comparison with long-duration GLP-1 RA trials is not appropriate. IKMT in participants with obesity significantly reduces sBP, dBP, mBP, PP, BW, and BMI from baseline. The best cases included 24 patients who achieved a WL from a BMI of 30 kg/m^2^ to a BMI < 23 kg/m^2^. The mean time to achieve this WL was 7.83 ± 2.54 (5–15) months, with a total treatment duration of 8.71 ± 2.46 (5–15) months. This exceeded the recommended duration of 6 months for EH; however, there were no significant AEs. At the 7-month follow-up examination, the WL remained substantial. The hypothesis that IKMT can be used in severe PwO requires confirmation in future prospective studies.

## Figures and Tables

**Table 1:** Baseline characteristics of the participants in each group.

Category	All Subjects (*n* = 3161)	Lose BW ≥ 10% (*n* = 1166)	Lose BW ≥ 5% (*n* = 2146)	Lose BW < 5% (*n* = 1015)
Age (yrs)	38.2 ± 11.39 (9–90) ≤10s (87) 20s (723) 30s (986) 40s (780) 50s (462) ≥60s (123)	30.81 ± 11.08 (9–73) ≤10s (41) 20s (244) 30s (368) 40s (321) 50s (157) ≥60s (35)	38.0 ± 11.2 (9–74) ≤10s (63) 20s (484) 30s (674) 40s (545) 50s (311) ≥60s (69)	38.6 ± 11.8 (12–90) ≤10s (24) 20s (239) 30s (312) 40s (235) 50s (151) ≥60s (54)
Gender	Female (2238, 70.8%); Male (923, 29.2%)	Female (850, 72.9%); Male (316, 27.1%)	Female (1525, 71.1%); Male (621, 29.9%)	Female (713, 70.2%); Male (302, 29.8%)
BW (kg)	91.99 ± 14.81 (62.0–185.9)	91.79 ± 14.24 (64.4–167.8)	92.04 ± 14.61 (64.2–181.8)	91.88 ± 15.23 (62.0–185.9)
Height (cm)	164.71 ± 8.82 (121.7–197.3)	164.13 ± 8.43 (139.7–189.5)	164.65 ± 8.74 (136.5–192.0)	164.84 ± 8.97 (121.7–197.3)
BMI (kg/m^2^)	33.78 ± 3.69 (30.0–63.9)	33.97 ± 3.66 (30.0–55.4)	33.83 ± 3.68 (30.0–63.9)	33.67 ± 3.72 (30.0–58.1)
Duration (day)	142.62 ± 104.92 (7–600)	191.46 ± 95.57 (28–563)	156.65 ± 99.58 (20–563)	112.97 ± 109.69 (7–600)

Categorical data are presented as frequencies and/or percentages. Continuous data are presented as means ± standard deviations (minimum–maximum values). Abbreviations: BW, body weight; BMI, body mass index.

**Table 2:** Baseline characteristics of the best-case participants (*n* = 24).

Category	Values
Age (yrs)	36.54 ± 11.64 (20–60) ≤10’s (0); 20’s (9); 30’s (7); 40’s (3); 50’s (4); ≥60’s (1)
Gender	Female (23, 95.8%); male (1, 4.2%)
BW (kg)	80.99 ± 7.28 (67.0–98.1)
Height (cm)	161.15 ± 7.08 (147.2–179.3)
BMI (kg/m^2^)	31.13 ± 0.97 (30.0–34.0)
Duration (day)	261.75 ± 75.31 (150–450)
Drinking	Drinking (12, 50%); none (12, 50%)
Menstruation	Menstruation (19, 79.17%); menopause (4, 16.67%)
Present illness	Edema (13); sleep disorder (12); gastrointestinal disease (9); constipation (8); urologic disease (2); allergic disease (2); liver disease (1); respiratory disease (1); mental illness (1)
Medication history	Hypoglycemic agent (2); antihypertensive drugs (2); anti-dyslipidemia agent (1); antipsychotics (1); analgesic (1); drugs for anemia (1); laxatives (1); steroids (2); antacids (1); hepatoprotective drugs (1); gout medications (1)
Family history	Diabetes mellitus (2); hypertension (1); dyslipidemia (1)
Treatment-experienced patient (for obesity)	HM (6, 25%); Western medicine (6, 25%); both (2, 8.33%); none (8, 33.33%)
Job	Service (7); office (5); unemployed (4); teaching (1); research (1); housewife (1), student (1); no information (4)

Categorical data are presented as frequencies and/or percentages. Continuous data are presented as means ± standard deviations (minimum–maximum values). Best cases: Weight loss with a BMI < 23 kg/m^2^. Abbreviations: BW, body weight; BMI, body mass index; HM, herbal medicine.

**Table 3:** Type of prescribed decoction herbal medicine.

Herbal Medicine Decoction	N
Fangji Huangqi-tang	5
Daeat-tang, Wuling Zhengqi-san	3
Danggui Shaoyao-san, Erchen Siwu-tang, Linggui zhugan-tang, Jingfang Xiebai-san	2
Jiawei Xiaoyao-san, Jiawei Changbai-san, Guizhi Fuling-wan, Banxia Baizhu Tianma-tang, Banxia Xiexin-tang, Chaihu Jialonggu Muli-tang, Wuling-san, Diaowei Chengqi-tang, Diaowei Shengqing-tang, Qingshang Juantong-tang, Qingxin Wendan-tang, Taiyin Diaowei-tang, Xiang Shapingwei-san, Xuanfu Lijing-tang	1

**Table 4:** Change in clinical outcomes (*n* = 3161).

	Baseline	Last (Endpoint)	Change	*p*-Value
BW (kg)	91.95 ± 14.78	83.94 ± 14.88	−8.02 ± 6.67	<0.001 *
TBW (kg)	38.94 ± 8.10	37.88 ± 8.01	−1.06 ± 1.64	<0.001 *
BFM (kg)	38.91 ± 8.44	32.32 ± 9.19	−6.59 ± 5.82	<0.001 *
BMI (kg/m^2^)	33.78 ± 3.69	30.82 ± 3.99	−2.96 ± 2.41	<0.001 *
BF% (%)	42.44 ± 6.37	38.42 ± 7.60	−4.02 ± 4.25	<0.001 *
WHR	0.98 ± 0.06	0.95 ± 0.07	−0.04 ± 0.05	<0.001 *
BI (%)	158.69 ± 17.33	144.81 ± 18.69	−13.88 ± 11.36	<0.001 *

* Statistically significant (*p* < 0.05). Paired *t*-tests (if data were normally distributed) were used. Continuous data are presented as means ± standard deviations. Abbreviations: BW, body weight; TBW, total body water; BFM, body fat mass; BMI, body mass index; BF%, body fat percentage; WHR, waist-to-hip ratio; BI, Broca’s index. The cut-off values were as follows: BMI, 23 kg/m^2^; WHR (male, 0.9; female, 0.85); BI, 120%.

**Table 5:** Change in clinical outcomes of best cases (*n* = 24).

	Baseline	Last (Endpoint)	Change	*p*-Value
BW (kg)	80.99 ± 7.28	57.97 ± 5.75	−23.02 ± 4.07	<0.001 *
TBW (kg)	33.35 ± 4.84	30.23 ± 4.47	−3.11 ± 1.34	<0.001 *
BFM (kg)	35.49 ± 3.67	16.78 ± 2.74	−18.71 ± 3.83	<0.001 *
BMI (kg/m^2^)	31.12 ± 0.97	22.24 ± 0.62	−8.90 ± 1.47	<0.001 *
BF% (%)	43.98 ± 4.18	29.15 ± 4.90	−14.83 ± 3.78	<0.001 *
WHR	0.96 ± 0.04	0.84 ± 0.04	−0.12 ± 0.05	<0.001 *
BI (%)	148.08 ± 4.93	105.67 ± 2.87	−42.42 ± 6.96	<0.001 *

* Statistically significant (*p* < 0.05). Paired *t*-tests (if data were normally distributed) were used. Continuous data are presented as means ± standard deviations. Abbreviations: BW, body weight; TBW, total body water; BFM, body fat mass; BMI, body mass index; BF%, body fat percentage; WHR, waist-to-hip ratio; BI, Broca’s index. The cut-off values were as follows: BMI, 23 kg/m^2^; WHR (male, 0.9; female, 0.85); BI, 120%.

**Table 6:** Change of blood pressure index.

Total subjects with before-and-after BP index comparisons (*n* = 1869)				
**BP Index**	**Baseline** **(*n* = 1869)**	**Last (Endpoint)** **(*n* = 1869)**	**Change** **(*n* = 1869)**	***p*** **-Value** **(*n* = 1869)**
sBP (mmHg)	139.70 ± 17.48	131.06 ± 16.40	−8.63 ± 16.33	<0.001 *
dBP (mmHg)	84.56 ± 14.25	78.17 ± 13.82	−6.39 ± 12.67	<0.001 *
mBP (mmHg)	102.61 ± 14.49	95.47 ± 13.76	−7.14 ± 12.71	<0.001 *
HR (bpm)	91.73 ± 13.75	99.84 ± 14.34	8.11 ± 14.47	<0.001 *
PP (mmHg)	55.13 ± 11.04	52.89 ± 11.14	−2.24 ± 12.47	<0.001 *
Best-case series subjects with before-and-after BP index comparisons (N = 21)				
**BP Index**	**Baseline** **(*n* = 21)**	**Last (Endpoint)** **(*n* = 21)**	**Change** **(*n* = 21)**	***p*** **-Value** **(*n* = 21)**
sBP (mmHg)	126.57 ± 12.28	110.62 ± 12.03	−15.95 ± 12.40	<0.001 *
dBP (mmHg)	75.81 ± 8.75	63.81 ± 13.19	−12.00 ± 13.39	<0.001 *
mBP (mmHg)	92.38 ± 9.28	79.10 ± 12.48	−13.29 ± 12.44	<0.001 *
HR (bpm)	94.67 ± 17.36	98.05 ± 15.12	3.38 ± 12.95	0.246
PP (mmHg)	50.76 ± 8.09	46.81 ± 5.65	−3.95 ± 8.36	0.042 *

* Statistically significant (*p* < 0.05), mean ± standard deviation. Paired *t*-tests (if data were normally distributed) were used. Three participants whose data were not comparable before and after the intervention were excluded. Abbreviations: BP, blood pressure; sBP, systolic blood pressure; dBP, diastolic blood pressure; mBP, mean blood pressure; HR, heart rate; PP, pulse pressure. All blood pressure indices were measured automatically using an InBody device. Of the 3161 patients, only 1869 had BP indices before and after the comparison. Only 21 of the 24 best cases showed BP indices before and after the comparison.

**Table 7:** Adverse events by system organ class and causality.

System-Organ Classes	Symptom	Values	Causality	
			Possible	Unlikely
Gastrointestinal system disorders	Constipation	17 (70.8)	0 (0)	17 (70.8)
	Nausea	7 (29.2)	3 (12.5)	4 (16.7)
	Mouth dry	6 (25)	0 (0)	6 (25)
	Esophageal pain	1 (4.2)	0 (0)	1 (4.2)
	Diarrhea	1 (4.2)	0 (0)	1 (4.2)
	Heartburn	1 (4.2)	0 (0)	1 (4.2)
Psychiatric disorders	Insomnia	4 (16.7)	1 (4.2)	3 (12.5)
Central and peripheral nervous system disorders	Dizziness	6 (25)	0 (0)	6 (25)
	Anxiety neurosis	1 (4.2)	0 (0)	1 (4.2)
	Headache	1 (4.2)	0 (0)	1 (4.2)
Body as a whole-general disorders	Fatigue	3 (12.5)	0 (0)	3 (12.5)
	Edema legs	2 (8.3)	2 (8.3)	0 (0)
	Chest discomfort	2 (8.3)	0 (0)	2 (8.3)
Musculoskeletal system disorders	Muscle pain	1 (4.2)	0 (0)	1 (4.2)
Skin and appendage disorders	Alopecia	1 (4.2)	0 (0)	1 (4.2)
Reproductive disorders	Menstrual irregularity	1 (4.2)	0 (0)	1 (4.2)

Values are presented as number (percentage) of patients in the safety analysis set (*n* = 24). Only the listed adverse events occurred; no other adverse events were reported.

**Table 8:** Contraindications in the Daeat Korean medical clinic.

Contraindications
(1) Liver disease (chronic hepatitis, cirrhosis, etc.)
(2) Heart disease (pacemaker, angina, heart failure, etc.)
(3) Brain disease (brain disease (cerebral hemorrhage, cerebral infarction that occurred within the last three months), brain aneurysm, epilepsy, etc.)
(4) Kidney disease (chronic kidney failure, kidney dialysis, etc.)
(5) Mental illness (schizophrenia, mentally agitated, taking some psychotropic medications (e.g., MAOIs), etc.)
(6) Closed-angle glaucoma
(7) Uncontrolled hyperthyroidism
(8) Taking certain medications (antifungals, TB medications, etc.)
(9) Waiting for an organ transplant or taking immunosuppressive medications after an organ transplant
(10) Immunocompromised individuals with AIDS
(11) Breastfeeding, pregnant women
(12) Professional athletes or occupational workers subjected to frequent doping tests
(13) BMI < 18.5 kg/m^2^, BW or BFM below the standard range

Abbreviations: MAOI, monoamine oxidase inhibitor; TB, tuberculosis; AIDS, acquired immunodeficiency syndrome; BMI, body mass index; BW, body weight; BFM, body fat mass.

**Table 9:** Composition of pill preparation (Daeat-dan) for 1 day.

No.	Ingredients	Scientific Name	Daily Dose (g)
1	Ephedrae Herba	*Ephedra equisetina* Bunge or *Ephedra intermedia* Schrenk et C. A. Meyer or *Ephedra sinica* Stapf	8.40
2	Coicis Semen	*Coix lacryma-jobi* Linné var. ma-yuen Stapf	0.90
3	Artemisiae Capillaris Herba	*Artemisia capillaris* Thunberg	0.90
4	Gardeniae Fructus	*Gardenia jasminoides* Ellis	0.90
5	Arecae Semen	*Areca catechu* Linné	0.45
6	Rehmanniae Radix Preparata	*Rehmannia glutinosa* Liboschitz ex Steudel	0.45
7	Puerariae Radix	*Pueraria lobata* Ohwi	0.45
8	Atractylodis Rhizoma Alba	*Atractylodes macrocephala* Koidzumi or *Atractylodes japonica* Koidzumi	0.45
9	Zingiberis Rhizoma	*Zingiber officinale* Roscoe	0.45
10	Poria Sclerotium	*Poria cocos* Wolf	0.45
11	Citri Unshiu Pericarpium	*Citrus unshiu* Markovich; Citrus reticulata Blanco	0.45
12	Magnoliae Cortex	*Magnolia officinalis* Rehder et Wilson var. biloba Rehder et Wilson or *Magnolia ovobata* Thunberg or *Magnolia officinalis* Rehder et Wilson	0.45
13	Ponciri Fructus Immaturus	*Poncirus trifoliata* Rafinesque	0.45
14	Sappan Lignum	*Caesalpinia sappan* Linné	0.45

## Data Availability

The data presented in this study are not publicly available due to privacy or ethical restrictions but are available from the corresponding author upon reasonable request. De-identified data are securely stored by the authors and can be shared in accordance with institutional and ethical guidelines.

## Abbreviations

The following abbreviations are used in this manuscript:

AEs	Adverse Events
AOM	Anti-Obesity Medication
BF%	Body Fat Percentage
BFM	Body Fat Mass
BIA	Bioelectrical Impedance Analysis
BI	Broca’s Index
BMI	Body Mass Index
BP	Blood Pressure
BW	Body Weight
dBP	Diastolic Blood Pressure
EH	Ephedrae Herba
FTF	Face-to-Face Treatment
GLP-1 RAs	Glucagon-like Peptide-1 Receptor Agonists
HM	Herbal Medicine
HR	Heart Rate
IKMT	Integrative Korean Medicine Treatment
KMC	Korean Medical Clinic
KMD	Korean Medicine Doctor
LM	Lifestyle Modification
MA	Medication Adherence
MCID	Minimal Clinically Important Difference
mBP	Mean Blood Pressure
NFTF	Non-Face-to-Face Treatment
NP	Network Pharmacology
PA	Pharmacopuncture
PK	Pharmacokinetic
PP	Pulse Pressure
PwO	People with Obesity
RCT	Randomized Controlled Trial
RWD	Real-World Data
sBP	Systolic Blood Pressure
TBW	Total Body Water
TEA	Thread Embedding Acupuncture
WHO	World Health Organization
WHR	Waist-to-Hip Ratio
WL	Weight Loss
